# The Cohesin Release Factor WAPL Restricts Chromatin Loop Extension

**DOI:** 10.1016/j.cell.2017.04.013

**Published:** 2017-05-04

**Authors:** Judith H.I. Haarhuis, Robin H. van der Weide, Vincent A. Blomen, J. Omar Yáñez-Cuna, Mario Amendola, Marjon S. van Ruiten, Peter H.L. Krijger, Hans Teunissen, René H. Medema, Bas van Steensel, Thijn R. Brummelkamp, Elzo de Wit, Benjamin D. Rowland

**Affiliations:** 1 Division of Cell Biology, the Netherlands Cancer Institute, Plesmanlaan 121, 1066 CX Amsterdam, the Netherlands; 2 Division of Gene Regulation, the Netherlands Cancer Institute, Plesmanlaan 121, 1066 CX Amsterdam, the Netherlands; 3 Division of Biochemistry, the Netherlands Cancer Institute, Plesmanlaan 121, 1066 CX Amsterdam, the Netherlands; 4 Hubrecht Institute, Uppsalalaan 8, 3584 CT Utrecht, the Netherlands; 5 Cancer Genomics Center, Plesmanlaan 121, 1066 CX Amsterdam, the Netherlands

**Keywords:** cohesin, CTCF, WAPL, SCC2, NIPBL, SCC4, MAU2, chromatin looping, TADs, loop extrusion

## Abstract

The spatial organization of chromosomes influences many nuclear processes including gene expression. The cohesin complex shapes the 3D genome by looping together CTCF sites along chromosomes. We show here that chromatin loop size can be increased and that the duration with which cohesin embraces DNA determines the degree to which loops are enlarged. Cohesin’s DNA release factor WAPL restricts this loop extension and also prevents looping between incorrectly oriented CTCF sites. We reveal that the SCC2/SCC4 complex promotes the extension of chromatin loops and the formation of topologically associated domains (TADs). Our data support the model that cohesin structures chromosomes through the processive enlargement of loops and that TADs reflect polyclonal collections of loops in the making. Finally, we find that whereas cohesin promotes chromosomal looping, it rather limits nuclear compartmentalization. We conclude that the balanced activity of SCC2/SCC4 and WAPL enables cohesin to correctly structure chromosomes.

## Highlights

• Hi-C analysis demonstrates that chromatin loop size can be increased genome-wide
• The duration with which cohesin embraces DNA determines the length of chromatin loops
• Haploid genetics reveals that the SCC2/SCC4 complex promotes loop extension
• Cohesin limits the compartmentalization of chromatin within the nucleus

## Graphical Abstract

**Figure:**
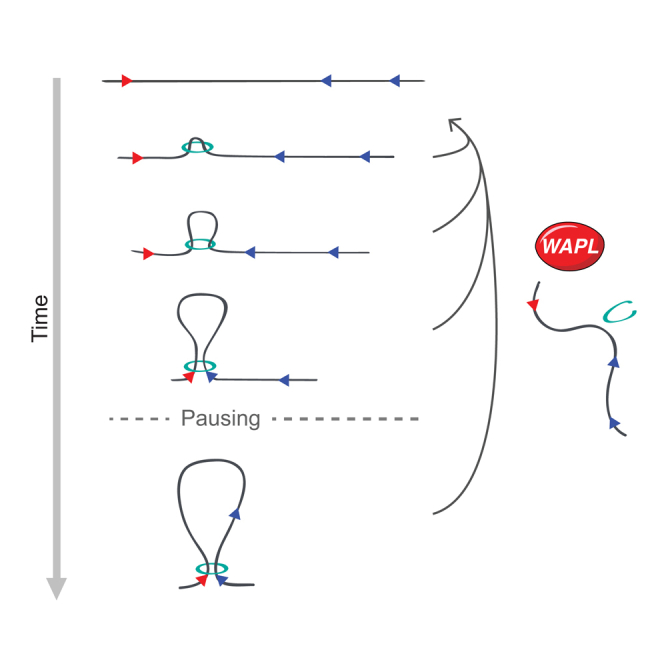

## Teaser

Cohesin's dynamic association with DNA determines the length of chromatin loops and allows this complex to correctly structure chromosomes.

## Introduction

Human chromosomes are linear stretches of DNA that are centimeters in length but are structured such that they fit into a nucleus with a diameter of a few micrometers. Within this confined space, DNA is organized to allow for key nuclear processes. The ring-shaped cohesin complex plays a major role in many aspects of chromosome biology, including sister chromatid cohesion, DNA repair, and transcriptional regulation. Cohesin by default transiently associates with DNA, which is thought to be the consequence of a continuous cycle of DNA entrapment and release (Haarhuis et al., 2014). Cohesin is loaded onto DNA by the SCC2/SCC4 complex (also known as NIPBL and MAU2, respectively), and DNA release is driven by cohesin’s antagonist WAPL (Ciosk et al., 2000, Gandhi et al., 2006, Kueng et al., 2006).

The cohesin complex consists of three core subunits, SMC1, SMC3, and SCC1 (also known as RAD21 or Mcd1), that together form a ring-shaped structure that can entrap DNA inside its lumen (Haering et al., 2008). WAPL drives cohesin’s release from chromatin by opening up a distinct DNA exit gate at the interface connecting cohesin’s SMC3 and SCC1 subunits (Beckouët et al., 2016, Murayama and Uhlmann, 2015). In the absence of WAPL, cohesin is loaded onto DNA, but does not turn over (Kueng et al., 2006, Tedeschi et al., 2013). WAPL-deficient interphase cells harbor an unusual granular DNA staining pattern and display a distinct nuclear thread-like cohesin distribution referred to as “vermicelli” (Tedeschi et al., 2013). This indicates that cohesin turnover at chromatin is somehow required to obtain the correct interphase chromosome organization.

On chromatin, cohesin colocalizes with the CCCTC binding factor CTCF (Parelho et al., 2008, Wendt et al., 2008). Together, cohesin and CTCF are key to the formation and/or maintenance of *cis* chromatin loops and create boundaries between topologically associated domains (TADs) (Merkenschlager and Nora, 2016). These domains are thought to reflect chromosomal regions that act as autonomous transcriptional units (Noordermeer et al., 2011). Recent work has shown that chromatin loops are formed almost exclusively between “convergent” CTCF sites (i.e., sites with consensus CTCF motifs pointing toward each other) (Rao et al., 2014, Vietri Rudan et al., 2015), and this specific orientation is required for the looping together of CTCF sites (de Wit et al., 2015, Guo et al., 2015, Sanborn et al., 2015). The molecular mechanisms controlling this “CTCF directionality looping rule,” however, remain unclear.

How chromatin loops are formed is one of the main outstanding questions in chromosome biology. One model is that cohesin entraps small loops inside its lumen, and the extrusion of such loops leads to the processive enlargement of loops up to often megabase-sized structures (Nasmyth, 2001). In this model (generally referred to as the “loop extrusion model”) (Alipour and Marko, 2012), CTCF limits the further extrusion, which is consistent with the presence of cohesin at CTCF sites and the requirement for the specific orientation of CTCF binding sites found in chromatin loops. Indeed, if cohesin during the looping process were to scan chromosomes in a linear manner, it may be able to detect the orientation of a CTCF site. Loop extrusion would also explain the organization of interphase chromosomes into TADs flanked by CTCF sites (Fudenberg et al., 2016). Here, we provide experimental evidence in support of the model that cohesin structures chromosomes through the processive enlargement of chromatin loops. We also show that the balanced activity of WAPL and the SCC2/SCC4 complex allows cohesin to correctly structure chromosomes.

## Results

### WAPL Restricts Chromatin Loop Extension

**Figure S1:**
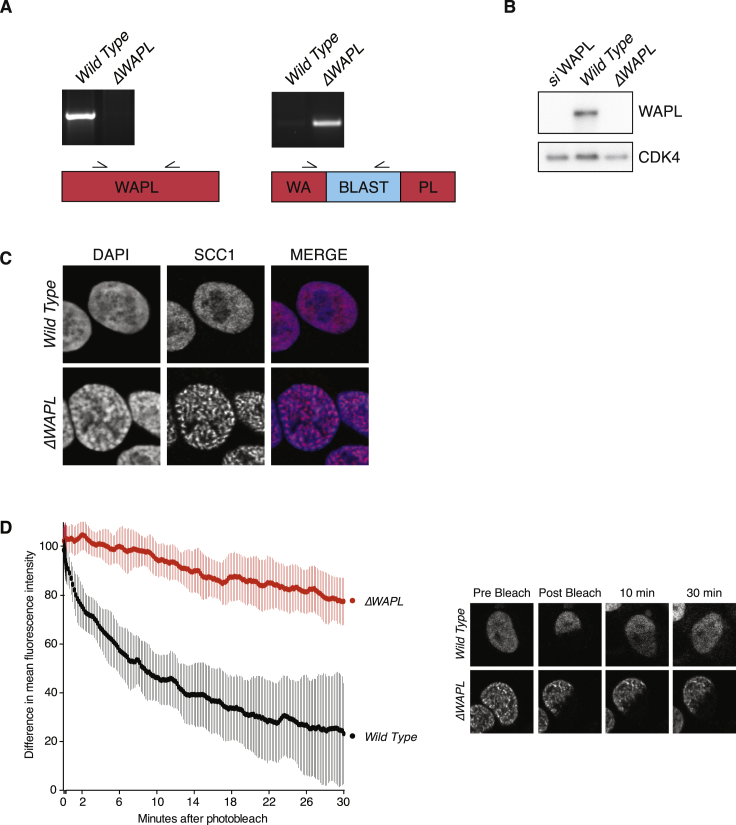
Characterization of *ΔWAPL* Cells, Related to Figures 1 and 3 (A) Genotype analysis of wild-type and *ΔWAPL* cells. (B) Western blot analysis of wild-type and *ΔWAPL* cells. WAPL siRNA-transfected cells are included as a control. (C) Immunofluorescence after pre-extraction of DNA-bound SCC1. (D) FRAP analysis of G1 cells expressing SCC1-GFP. Difference between non-bleached and bleached regions is plotted, including representative images of the FRAP movies (wild-type n = 7, *ΔWAPL* n = 6). The FRAP plots in Figure 3I include the same data and Figure S5B shows the bleaching control.

To test whether cohesin-mediated DNA looping requires cohesin’s turnover on chromatin, we generated WAPL knockout HAP1 cells using CRISPR technology. As expected, WAPL deficiency severely impaired cohesin’s turnover on chromatin, led to a marked increase of cohesin’s association at DNA, and yielded cells that displayed the vermicelli thread-like cohesin staining pattern (Figure S1). It is important to note for our further analyses that HAP1 cells proliferated normally in the absence of WAPL, likely due to the fact that these cells have impaired p53 function (Haarhuis et al., 2013).

**Figure 1:**
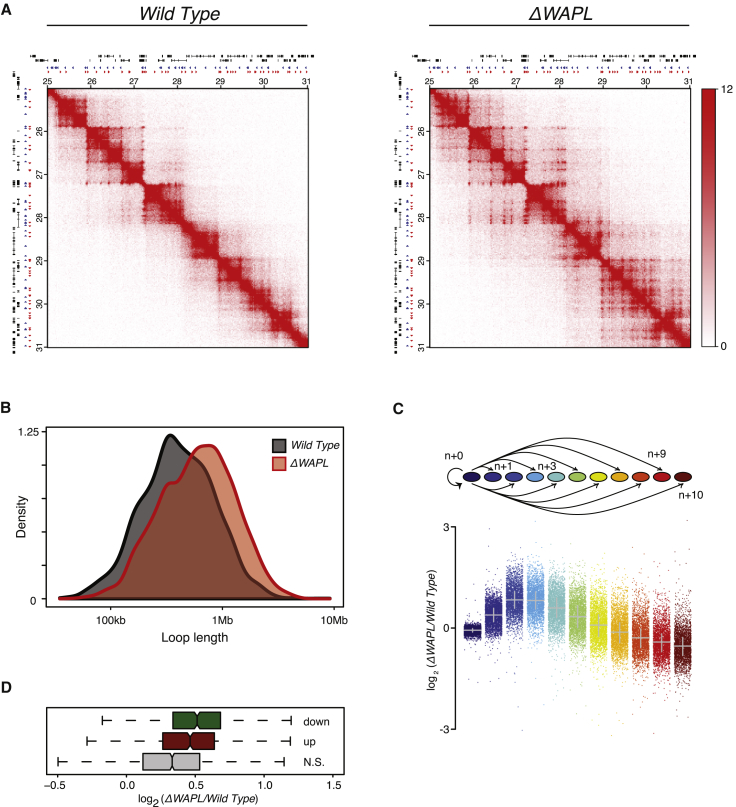
WAPL Restricts Chromatin Loop Extension (A) Hi-C contact matrices for a zoomed in region on chromosome 7. Contact matrices are normalized to 100 million contacts, shown resolution is 20 kb. Above and to the left of the contact matrices the union of CTCF sites identified in wild-type and *ΔWAPL* are shown. Red and blue triangles denote forward and reverse CTCF sites, respectively. (B) Density plot showing the length distribution of the loops called by HICCUPS (Rao et al., 2014) in wild-type and *ΔWAPL* cells. (C) Contact frequency analysis of a given TAD and its ten flanking TADs. The log2-ratio of the contact frequency between two TADs in *ΔWAPL* over wild-type is plotted. (D) Quantification of the difference in contact frequency of directly neighboring TADs (n+1) between wild-type and *ΔWAPL*. TADs are stratified into those that contain downregulated promoters or upregulated promoters, or TADs containing promoters that show no significant (N.S.) difference in expression. Wilcoxon rank-sum test shows a significant increase in contact frequency between TADs that contain an upregulated or downregulated promoter and TADs that do not contain a significantly affected promoter (p value upregulated genes = 8.40 × 10e−23, downregulated genes = 3.90 × 10e−44). See also Figures S1 and S2.

To study the role of WAPL in chromosome organization, we generated high-resolution Hi-C profiles (Rao et al., 2014) in control and *ΔWAPL* HAP1 cells. This method allows the visualization of chromatin interactions across the genome. In control cells, we observed looped-together CTCF sites that are visualized as relatively isolated dots off the Hi-C diagonal and TADs (domains that are enriched for *cis* interactions throughout) flanked by CTCF sites (Figure 1A, left).

**Figure S2:**
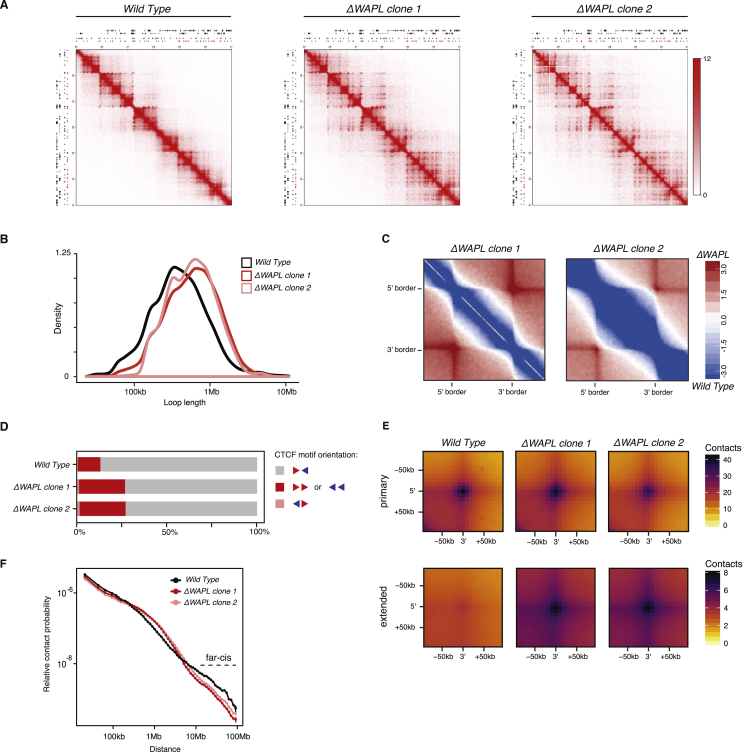
The *ΔWAPL* Phenotype Is Also Observed in the Biological Replicate, Related to Figures 1, 2, and 5 (A) Hi-C contact matrices for a zoomed in region on chromosome 7 for wild-type, *ΔWAPL* clone 1 and *ΔWAPL* clone 2. Contact matrices are normalized to 100 million contacts, shown resolution is 20kb. Above and to the left of the contact matrices the union of CTCF sites identified in wild-type and *ΔWAPL* are shown. Red en blue triangles denote forward and reverse CTCF sites, respectively. (B) Density plot showing the length distribution of the loops called by HICCUPS (Rao et al., 2014) in wild-type and *ΔWAPL* clone 1 and *ΔWAPL* clone 2. (C) The differential ATA signal between both biological replicates of *ΔWAPL* and wild-type is visualized for all TADs. Blue indicates a higher signal in the wild-type, red indicates a higher signal in *ΔWAPL* cells. (D) Quantification of the unique orientation of CTCF sites that could be associated with Hi-C loops called by HICCUPs. (E) APA for primary and extended loops. (F) Relative contact probability plot shows the likelihood of a contact at increasing length scales. Error bars depict the standard error of the mean.

Intriguingly, WAPL deficiency led to the extension of chromatin loops and strongly increased interaction frequencies between nearby TADs (Figures 1A–1C and S2A). When we performed systematic loop calling on the full-genome contact maps (Rao et al., 2014), we found that the median loop length increased by more than 200 kb in the *ΔWAPL* cells compared to the wild-type (575 kb versus 370 kb). These results show that cohesin turnover is not required for the formation of TADs and loops, but rather restricts the length of loops and intra-TAD interactions. The size of chromatin loops apparently can be increased, and the duration with which cohesin embraces DNA determines the degree to which loops are enlarged.

**Figure S3:**
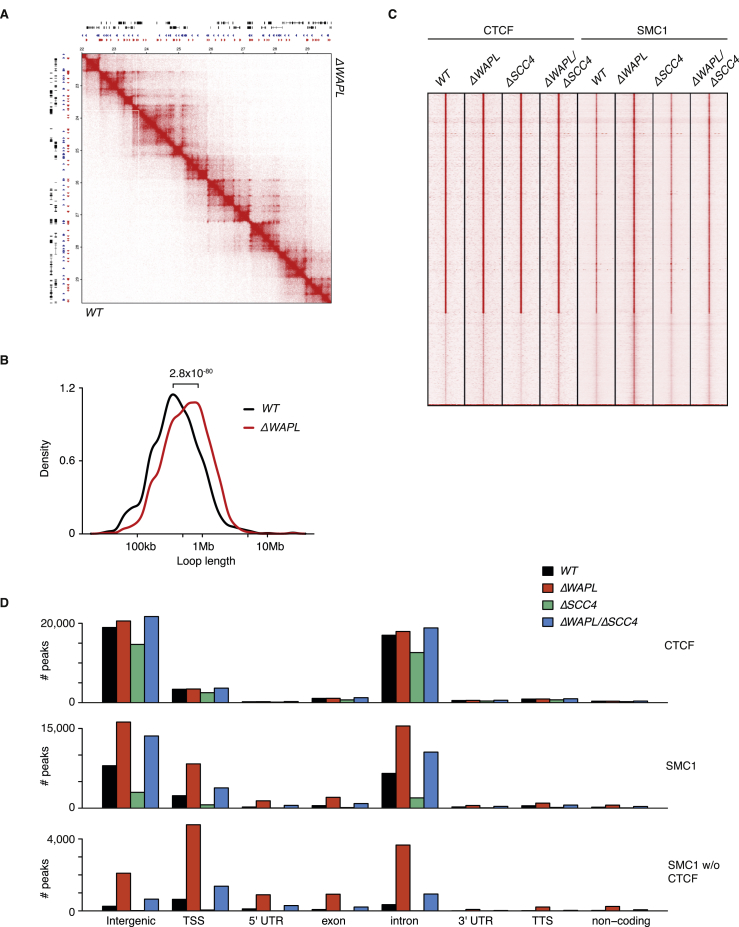
Genomic Regions Unaffected in Gene Expression Also Display Increased Loop Length, Related to Figure 3 (A) Comparative heatmap of a genomic region that does not contain any differentially regulated genes. Bottom triangle is the wild-type Hi-C data, the top triangle is the *ΔWAPL* data. (B) Density plot of the loop length in genomic blocks (at least 4Mb) without differentially expressed genes. (C) ChIPseq heatmap of CTCF and SMC1 (cohesin) in WT, *ΔWAPL*, *ΔSCC4* and *ΔWAPL/ΔSCC4* for SMC1 binding sites in *ΔWAPL* cells. (D) Annotation of ChIP peaks of CTCF (top panel), all SMC1 peaks (middle panel) and non-CTCF SMC1 peaks (bottom panel).

The formation of loops and TADs has been suggested to be linked to gene expression (Giorgetti et al., 2016, Rao et al., 2014), although the causal relationship between them has remained unresolved. We analyzed gene expression in control and *ΔWAPL* cells and found that 1,009 genes were differentially expressed. One possibility is that local gene expression influences loop extension. However, when we looked at regions that harbor no differentially expressed genes we found that also in these regions loop length is increased (Figures S3A and S3B), showing that gene expression differences are not a global explanation for the increase in loop length. Similarly, we observed a global increase in the interaction frequency between most neighboring TADs in *ΔWAPL* cells. However, those TADs that contained differentially expressed genes showed significantly stronger interactions (Figure 1D), suggesting that the aberrant interaction between TADs can affect the expression of genes within these domains.

### WAPL-Deficient Cells Accumulate Contacts at TAD Corners

The defining feature of TADs is that they have more intra-domain interactions than with the surrounding sequence, as such they are visible as squares in the Hi-C contact matrix. One interesting possibility is that TADs reflect a polyclonal collection of loops in the making between two CTCF sites, and the frequent disassembly of loops followed by their reforming ensures that TADs remain dynamic (Fudenberg et al., 2016). If loops were formed through the processive enlargement of small loops, and if TADs are indeed the product of different degrees of incomplete loops between two CTCF sites, then the stochastic removal of cohesin from DNA by WAPL would be one way to achieve dynamic TAD-like chromosomal structures. In that case, WAPL deficiency should affect the distribution of contacts within a TAD and should primarily yield loops spanning the maximal distance within a TAD.

**Figure 2:**
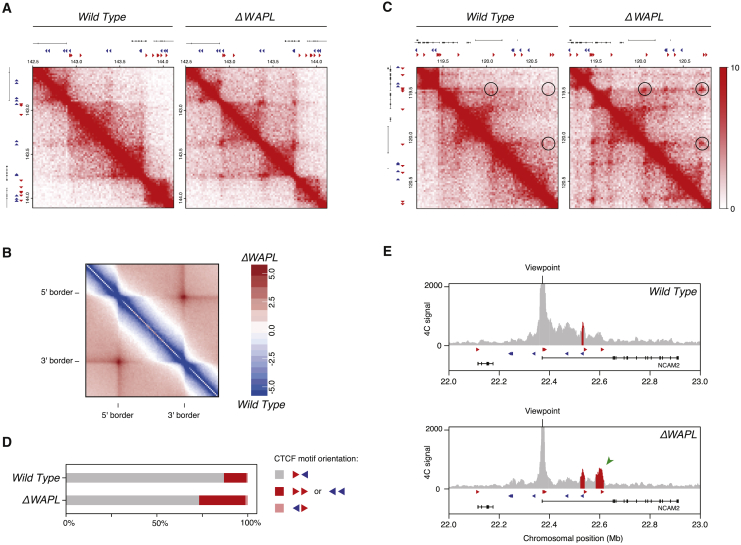
WAPL-Deficient Cells Accumulate Contacts at TAD Corners (A) Hi-C contact matrices for a zoomed in region on chromosome 2 similar to Figure 1A. Forward and reverse CTCF sites are depicted as red and blue triangles, respectively. (B) Aggregate TAD analysis (ATA) calculates the average Hi-C signal across a selected set of TADs. The differential ATA signal between *ΔWAPL* and wild-type is visualized for all TADs in the size range 500 kb–1 Mb. Blue indicates a higher signal in the wild-type, red indicates a higher signal in *ΔWAPL* cells. (C) Examples of illegally oriented chromosomal loops. Contact matrices similar to Figure 1A. Red and blue triangles denote forward and reverse CTCF sites, respectively. (D) Quantification of the unique orientation of CTCF sites that could be associated with Hi-C loops called by HICCUPs. (E) 4C-seq analysis for a CTCF site in the NCAM2 locus. Viewpoint is indicated above graph. Red bars highlight regions identified significantly above background (“peaks”). Peak calling was performed with peakC. Green arrowhead depicts a specific interaction in *ΔWAPL* cells. Red and blue triangles show forward and reverse CTCF sites. See also Figures S2 and S7.

To test this hypothesis, we analyzed the fate of these structures in *ΔWAPL* cells. Importantly, we often found TADs that developed pronounced corner-peaks, i.e., peaks at the intersection of the 5′ and 3′ boundary of a TAD, in *ΔWAPL* cells (Figure 2A). When we calculated the average contact profile of the TADs that we identified in wild-type cells, we observed that this was a global effect (Figures 2B and S2C). The increase in signal at the corners of TADs was accompanied by a decrease in the intra-TAD contact frequency. Together, our results support the model that chromatin loops are formed through the processive enlargement of smaller loops, and indeed, TADs reflect polyclonal populations of loops in the making between two given CTCF sites. We suggest that WAPL-mediated cohesin release allows TADs to remain dynamic.

### WAPL Enforces the Specificity of Chromosomal Loops

To characterize the stabilization of cohesin on DNA following the knocking out of WAPL, we performed chromatin immunoprecipitation followed by sequencing (ChIP-seq) for CTCF and the cohesin subunit SMC1 in wild-type and *ΔWAPL* HAP1 cells. Our results show a strong increase in the number of SMC1 peaks following stabilization (from 18,462 to 45,479). In the *ΔWAPL* cells, we find two main classes of cohesin binding sites in: (1) peaks overlapping with CTCF sites, and (2) non-CTCF-associated cohesin sites that localize mostly around transcriptional start sites (TSSs) (Figure S3D). We see a clear increase in the binding signal for both classes of peaks.

Based on the CTCF ChIP data, we assigned an orientation to the loop anchors and found that wild-type HAP1 cells almost exclusively looped together convergent CTCF sites (Figures 2C–2E). However, when we characterized the chromatin loops in *ΔWAPL* cells, we found many examples of loops that violated the CTCF orientation rule (Figure 2C). We then systematically quantified the orientation of all loops that could be unambiguously assigned to two CTCF sites in control and *ΔWAPL* cells. Strikingly, we found an increase in tandem CTCF orientations in *ΔWAPL* cells compared to the wild-type (Figures 2D and S2D).

We confirmed these findings using 3C combined with sequencing (4C-seq) as an independent assay. This method enables the high-resolution identification of interactions with a specific genomic region or viewpoint. For example, at the *NCAM2* locus, which is strongly upregulated in *ΔWAPL* cells, we found that WAPL-deficient cells exhibited a loop connecting CTCF sites in the tandem orientation (Figures 2E and S7A ). Therefore, WAPL not only limits the extension of DNA loops per se but also counteracts the formation or maintenance of certain DNA loops.

### WAPL Deficiency Compensates for Impaired SCC2/SCC4 Function

**Figure 3:**
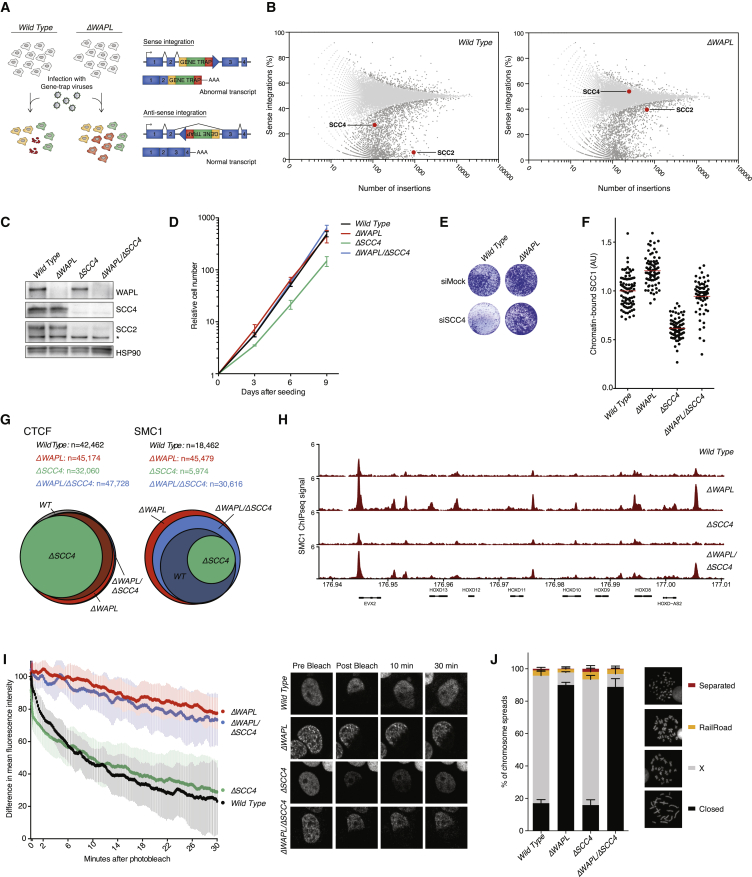
WAPL Deficiency Compensates for Impaired SCC2/SCC4 Function (A) Schematic overview of the haploid genetic screening set-up as used in (B). (B) The ratio of sense and antisense orientation insertions (y axis) in individual genes in wild-type and *ΔWAPL* cells. *SCC2* and *SCC4* are highlighted in red. The total number of insertions in the respective gene is plotted on the x axis. (C) Western blot analyses of the indicated cell lines. (D) 3T3-like proliferation curves of the indicated cell lines. Line shows the average and SDs of three experiments. (E) Colony formation assays of wild-type and *ΔWAPL* cells on transfection with small interfering RNAs (siRNAs) targeting *SCC4* or a control siRNA. (F) Quantitative immunofluorescence of chromatin-bound cohesin. Cells are pre-extracted to remove the unbound cohesin fraction. Intensity of remaining SCC1 is measured. Each dot depicts the signal per cell, red line indicates the mean and n is at least 75 cells per sample. (G) Venn diagram showing the overlap of CTCF and cohesin (SMC1) bound sites, assessed by chromatin immunoprecipitation (ChIP). (H) Examples of ChIP profiles as used in (G). (I) FRAP analysis of G1 cells expressing SCC1-GFP. Difference between non-bleached and bleached regions is plotted, including representative images of the FRAP movies (wild-type n = 7, *ΔWAPL* n = 6, *ΔSCC4* n = 10, *ΔWAPL/ΔSCC4* n = 6). The FRAP plots in Figure S1 include the same data for wild-type and *ΔWAPL* cells and Figure S5B shows the bleaching control. (J) Chromosome spreads of the indicated cell lines, including examples of each scoring category. Average of three experiments, n is at least 100 spreads, error bar depicts SD. See also Figures S1, S3, and S5.

In order to identify key factors that rather promote DNA looping, we designed a genetic screen. We reasoned that looping factors are important for cellular viability, and that in the absence of WAPL, factors that promote looping may become less important. Our rationale was that due to cohesin’s strongly increased residence time on DNA, WAPL-deficient cells have more time to form their loops. Any defect in loop formation may thereby be compensated for by WAPL deficiency. We therefore performed a synthetic viability screen (Blomen et al., 2015), comparing control HAP1 cells to *ΔWAPL* HAP1 cells. This screening system entails the polyclonal infection of both cell lines with gene-trap viruses. As these cells are haploid, a single insertion in essence creates a knockout of the affected gene. If a gene is important for viability, cells harboring disruptive intronic insertions in the sense orientation of this gene will be depleted from the population over time (Figure 3A).

To our surprise, we found that *ΔWAPL* cells tolerated gene-trap insertions in the genes encoding both subunits of the SCC2/SCC4 cohesin loader complex (Figure 3B), while in wild-type cells, SCC2 and SCC4 displayed strong essentiality for fitness. When we generated SCC4 knockout cells by CRISPR technology, these turned out to be viable but impaired in growth. Notably, *ΔSCC4* cells displayed severely reduced SCC2 levels (Figure 3C), presumably due to reduced SCC2 stability in the absence of its binding partner (Hinshaw et al., 2015, Watrin et al., 2006). We validated that in a WAPL-deficient background, loss of SCC4 had no effect on proliferation (Figures 3D and 3E).

### SCC4-Independent Cohesin Loading

To measure the effect of SCC4 deficiency on the abundance and dynamics of cohesin on DNA, we performed both cytological and molecular analyses. Using immunofluorescence microscopy, we found a reduced amount of chromatin-associated cohesin (Figure 3F), and ChIP-seq analysis showed a more than 3-fold reduction in the amount of detected cohesin binding sites (from 18,462 peaks in wild-type to 5,974 in *ΔSCC4*) (Figure 3G). Finally, fluorescence recovery after photobleaching (FRAP) experiments showed that, although the amount of cohesin on DNA was reduced, its stability on chromatin was similar to wild-type cells (Figures 3I and S5B ). However, the reduced amount of cohesin on chromatin did not lead to an evident sister chromatid cohesion defect (Figure 3J). This latter finding is in correspondence with the earlier report that budding yeast requires only very little DNA-bound cohesin for sister chromatid cohesion (Heidinger-Pauli et al., 2010).

The co-depletion of WAPL restored the amount of cohesin on DNA to wild-type levels (Figure 3F). However, *ΔWAPL*/*ΔSCC4* cells displayed very stably DNA-bound cohesin that was similar to *ΔWAPL* cells (Figure 3I). This was also reflected in the amount of peaks identified by ChIP-seq analysis, which showed an increase of more than 12,000 peaks compared to wild-type. This higher number of cohesin-bound sites may be a readout of cohesin’s sliding along chromatin, which presumably requires stable DNA binding. Our finding that SCC4 is not strictly required for cohesin loading in vivo is surprising, as Scc4 is essential for loading and viability in budding yeast. It implies that human SCC4 aids cohesin in the loading reaction, but cohesin can, to some degree, load onto DNA without SCC4. This notion is in line with the recent finding that in vitro, cohesin can entrap DNA without its loader, albeit inefficiently (Murayama and Uhlmann, 2014, Stigler et al., 2016).

### The C-Terminal Part of SCC2 Drives the Formation of Vermicelli Chromosomes

**Figure 4:**
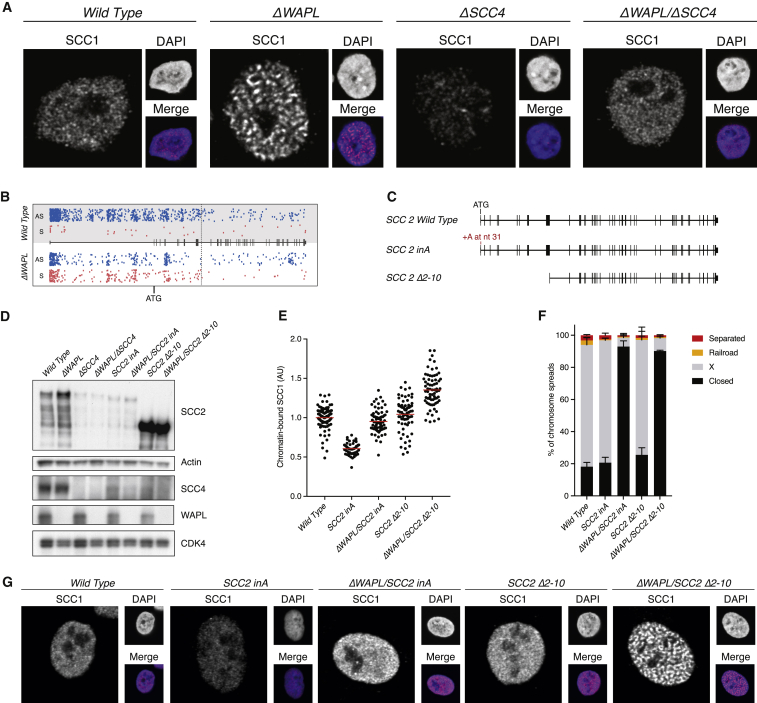
The C Terminus of SCC2 Drives the Formation of Vermicelli Chromosomes (A) Immunofluorescence after pre-extraction of DNA-bound SCC1. The vermicelli phenotype is clearly visible in *ΔWAPL* cells. (B) Gene-trap insertion patterns in sense (red) or anti-sense (blue) orientation in wild-type and *ΔWAPL* cells. (C) Schematic depiction of SCC2-truncation mutants. “*SCC2 inA*” cells harbor a frameshift mutation due to the insertion of an A after 31 nucleotides. “*SCC2 Δ2*-*10*” cells lack exons 2–10. (D) Western blot depicting expression of the indicated proteins. The SCC2 blot was generated using a C-terminal antibody with actin as a loading control. CDK4 is the loading control for the SCC4 and WAPL blots. (E) Quantitative immunofluorescence of chromatin-bound cohesin. Performed as in Figure 3F. n is at least 50, and the red line indicates the mean. (F) Chromosome spreads of the indicated cell lines. Scoring was performed as in Figure 3J. Average of three experiments, n is at least 100 spreads, error bar depicts SD. (G) Immunofluorescence after pre-extraction of DNA-bound SCC1. The vermicelli phenotype is clearly visible in *ΔWAPL*/*SCC2 Δ2*-*10* cells.

We then studied cohesin staining patterns and found that whereas *ΔWAPL* cells clearly displayed the vermicelli cohesin-staining phenotype, these thread-like structures were absent in *ΔWAPL*/*ΔSCC4* cells (Figure 4A). Apparently, SCC4 is required for the formation of vermicelli. To investigate how SCC4 contributes to this intriguing phenotype, we wished to test the effect of deleting SCC4’s binding partner SCC2. Remarkably, we were unable to knock out SCC2, even in a *ΔWAPL* background. How then could we identify SCC2 with our gene-trap screen for factors that are no longer required in the absence of WAPL? We therefore more closely investigated the insertion sites of the gene-trap viruses. We found that *ΔWAPL* cells only tolerated disruptive insertions up to and including the tenth exon of *SCC2* (Figure 4B). This would indicate that the region containing exons 11–47 remains essential even in *ΔWAPL* cells.

To mimic a gene-trap insertion early in SCC2, we disrupted the SCC2 reading frame after its ATG by CRISPR-mediated mutagenesis (Figure 4C). This led to loss of expression of full length SCC2 and yielded a shorter SCC2 product that lacked the SCC4 binding domain (Braunholz et al., 2012). This truncation was considerably less abundant than full length SCC2 was in the parental cells, and it also led to a decrease in SCC4 levels (Figure 4D). This presumably indicates that both SCC2 and SCC4 are less stable when they cannot form a heterodimer. Notably, the SCC2 mutation led to the same phenotype as did SCC4 deletion, namely a reduction of SCC1 binding to chromatin, both in wild-type and in a *ΔWAPL* background, to a loss of the vermicelli cohesin staining pattern of *ΔWAPL* cells, and these cells did not display a sister chromatid cohesion defect (Figures 4E–4G).

We then deleted the coding sequence up to exon 11 (Figure 4C). This yielded a truncated SCC2 protein that lacked a large N-terminal region including its SCC4-binding domain and again led to reduced SCC4 levels (Figure 4D). Importantly, this truncated SCC2 product was highly abundant, which would indicate that the deleted N-terminal region harbors a sequence that, when unbound to SCC4, destabilizes SCC2. This abundant truncation mutant was useful, as it allowed us to uncouple reduced SCC4 levels from SCC2 levels. We found that these SCC2-truncated cells, despite their reduced SCC4 levels, harbored normal SCC1 levels on chromatin, displayed robust sister chromatid cohesion, and importantly, also displayed vermicelli cohesin stainings in a *ΔWAPL* background (Figures 4E–4G).

This result indicates that the formation of vermicelli chromosomes requires the function of the C-terminal part of SCC2. It also indicates that the effect of SCC4 deletion on cohesin loading is not due to an inherent function of SCC4, but rather is a consequence of the severely reduced levels of SCC2 in *ΔSCC4* cells. The key role of the SCC2/SCC4 complex in cohesin loading and in the formation of vermicelli chromosomes apparently is mediated by the C-terminal part of SCC2. Notably, this is the most conserved part of SCC2. As SCC4 deletion results in loss of SCC4 and near-complete loss of SCC2 levels, we used SCC4-deficient cells to further study the role of the SCC2/SCC4 complex in chromosome organization.

### The SCC2/SCC4 Complex Promotes Loop Extension

**Figure 5:**
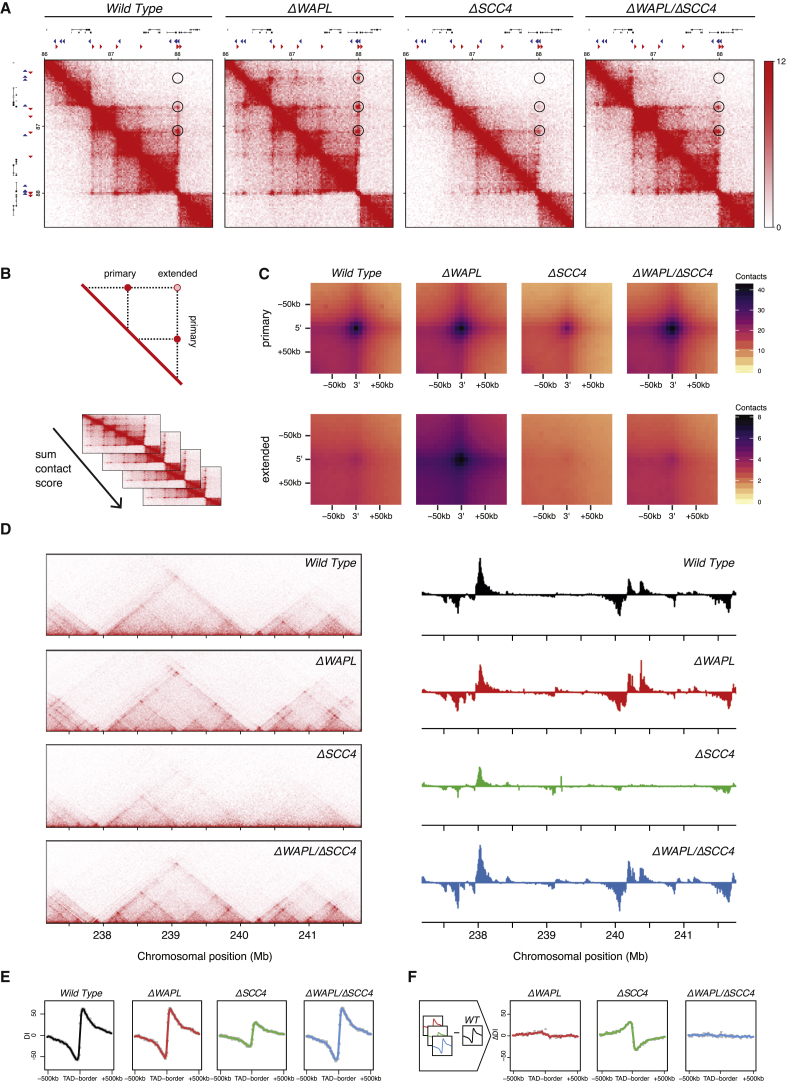
The SCC2/SCC4 Complex Promotes Loop Extension (A) Hi-C contact matrices for a zoomed in region on chromosome 5 similar to Figure 1A. Forward and reverse CTCF sites are depicted as red and blue triangles, respectively. (B) Top: schematic explaining the difference between primary and extended loops (see the STAR Methods for exact definition). Bottom: schematic explaining aggregate peak analysis (APA). (C) APA for primary and extended loops. (D) Heatmaps horizontally visualizing the Hi-C matrix along a zoomed in region on chromosome 1 for wild-type, *ΔWAPL*, *ΔSCC4*, and *ΔWAPL/ΔSCC4* cells (left). The four panels on the right plot the Directionality Index (Dixon et al., 2012) for the same region. (E) Directionality Index (DI) is calculated for the 100 kb up- and downstream of all 5′ TAD borders identified in wild-type HAP1 cells. Average profiles of the aligned directionality indices are plotted for the four cell lines. (F) Differential profiles of the aligned directionality indices (ΔDI) are plotted as the wild-type signal subtracted from the three knockout cell lines. See also Figures S2 and S6.

To investigate the role of the SCC2/SCC4 complex in DNA looping, we performed high-resolution Hi-C in *ΔSCC4* and *ΔWAPL*/*ΔSCC4* cells. We found that SCC4 loss led to shorter loops in a wild-type background and also reduced loop length in *ΔWAPL* cells (Figure 5A). Whereas WAPL restricts the extension of loops, the SCC2/SCC4 complex apparently promotes loop extension. We quantified this by performing a systematic analysis of loops. To this end, we performed aggregate peak analysis (APA) on primary loops (i.e., loops found in wild-type cells) and extended loops, which are predicted loops of increased length based on the wild-type loop anchors (Figure 5B). The APA confirmed our observation that extended loops are formed more frequently in *ΔWAPL* cells and showed that extended loops are mostly formed between existing loop anchors, rather than being formed between de novo anchors. In addition, we found that SCC4 deficiency caused a reduction in contact frequency between loop anchors identified in wild-type cells. SCC4 loss in *ΔWAPL* cells also led to a reduction in extended loops, resulting in an overall loop length that strongly resembled those found in wild-type cells (Figure 5C). Together, these findings indicate that the SCC2/SCC4 complex contributes to the extension of DNA loops.

Our Hi-C data also revealed that TADs in *ΔSCC4* cells displayed more diffuse boundaries (Figure 5D, left). We then calculated the directionality index (DI), which is a measure for TAD partition, and found that *ΔSCC4* cells indeed displayed severely decreased DI scores (Figure 5D, right). To quantify this on a genome-wide level, we aligned the DI for all TAD boundaries, which revealed that the more diffuse boundaries in *ΔSCC4* cells were a global effect, and this defect was rescued in *ΔWAPL*/*ΔSCC4* cells (Figures 5E, 5F, and S6D ).

Considering that the SCC2/SCC4 complex contributes to the extension of chromatin loops, and it is also required for the formation of defined TAD boundaries, these two phenotypes are presumably functionally linked. If a TAD de facto is a collection of loops in the making between two CTCF sites, then we envisage that in *ΔSCC4* cells, these loops only rarely extend far enough to reach one or both CTCF sites. Together, our data again support the model that cohesin structures chromosomes through the processive enlargement of loops, and indeed, TADs are collections of loops in the making between two given CTCF sites.

### Vermicelli Chromosomes Harbor Reduced Far-*cis* Contacts

**Figure 6:**
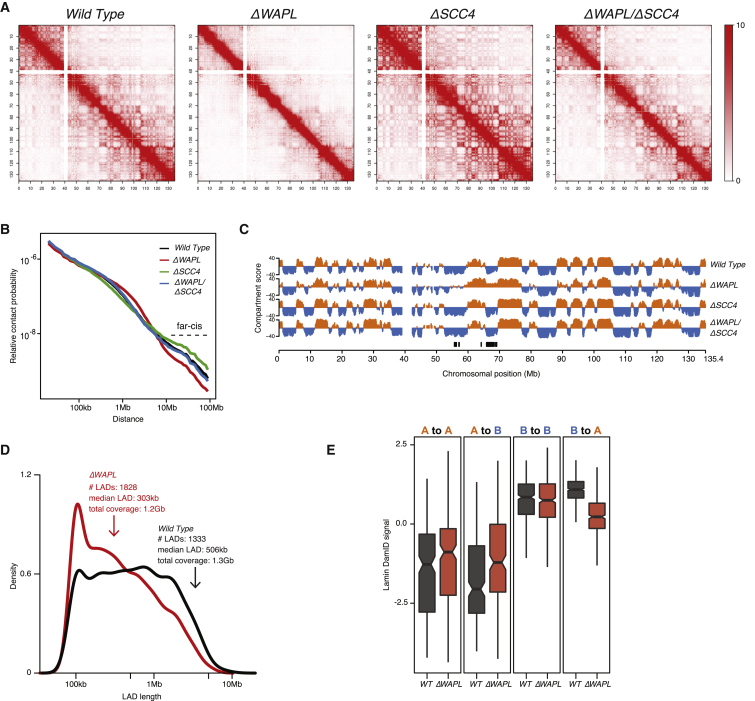
Vermicelli Chromosomes Harbor Reduced Far-*cis* Contacts (A) Whole-chromosome contact matrices for chromosome 10. Matrices are normalized to 100 million contacts, resolution shown is 150 kb. (B) Relative contact probability plot shows the likelihood of a contact at increasing length scales. (C) Compartment scores (see the STAR Methods) show the segregation of chromosome 10 into A and B compartments. Regions that switch from A to B or from B to A are highlighted below the graph (see the STAR Methods for definition of switching regions). (D) Density plots showing length distribution of LADs. For more detailed information, see Figure S4A. (E) Quantification of LaminB1 DamID signal (as a proxy for association of DNA to the nuclear periphery) for regions that switch compartment and regions that do not switch compartment. See also Figures S4 and S6.

Next, we studied the macro-scale consequences on chromosome architecture upon inactivation of WAPL, SCC4, or both. When we compared the whole-chromosome contact maps for wild-type and *ΔWAPL* cells, we saw a striking decrease in the number of far-*cis* (>10 Mb) contacts in the *ΔWAPL* cells. (Figure 6A). This is confirmed by calculating the contact probability over a given distance (Lieberman-Aiden et al., 2009), which is a robust statistic for the macromolecular organization of chromosomes (Figures 6B and S2F).

Conversely, *ΔSCC4* cells displayed an increase in far-*cis* contacts compared to wild-type cells (Figures 6A and 6B). As SCC4-deficient cells have less cohesin on chromatin, this result, together with the inverse phenotype of *ΔWAPL* cells, would suggest that cohesin restricts the flexibility of chromosomes. Remarkably, contact maps of *ΔWAPL*/*ΔSCC4* cells were very similar to those of wild-type cells (Figures 6A and 6B), indicating the SCC2/SCC4 complex and WAPL balance out each other’s functions regarding macro-scale chromosome organization.

### Cohesin Limits Nuclear Compartmentalization

**Figure S4:**
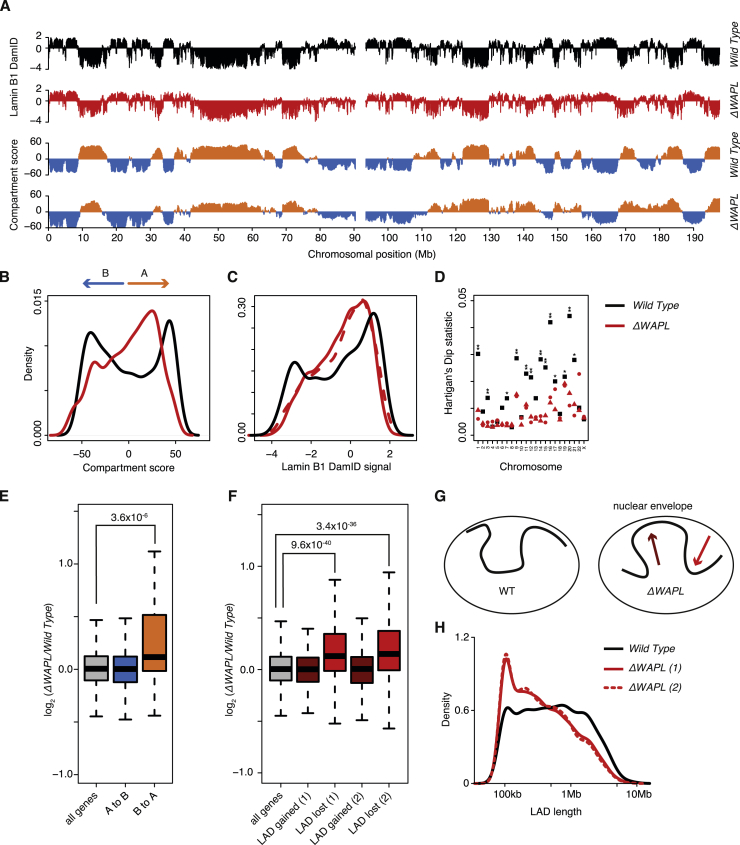
WAPL Deletion Restricts Nuclear Compartmentalization, Related to Figure 6 (A) Chromosomal map of DamID of LaminB1 and compartment scores for the wild-type and *ΔWAPL* cells for chromosome 3. (B) Density plot showing the distribution of compartment scores for chromosome 3. (C) Idem, but for the LaminB1 DamID signal, the solid and dotted line denote different *ΔWAPL* clones. (D) The Hartigan’s dip statistic, measuring bimodality of the LaminB1 DamID signal, is shown for all chromosomes. Black squares show the wild-type scores, red triangles and dots show the scores for the *ΔWAPL cells*. ^∗^ denotes multiple hypothesis corrected p value < 0.01, ^∗∗^p < 0.001. (E) Expression comparison for all genes that switch from A to B compartment and vice versa in *ΔWAPL* cells. (F) Idem, but for genes that switch from iLAD to LAD (dark red) and from LAD to iLAD (light red) in *ΔWAPL* cells. (1) and (2) denote different *ΔWAPL* clones. (G) Cartoon depicting shifts to and from the nuclear lamina as scored in the expression comparison in (F). (H) Density plot showing length distribution of LADs in wild-type and two *ΔWAPL* clones.

Regular interphase chromosomes segregate active and inactive genomic regions into A and B compartments, respectively (Lieberman-Aiden et al., 2009). These compartments are evident from the plaid-pattern of far-*cis* contacts on whole-chromosome Hi-C maps. Although they are less clearly defined in *ΔWAPL* cells, we can to some degree identify A and B nuclear compartments and they generally overlap with those found in wild-type, but the distribution of the scores is not as strongly bimodal as in the wild-type. This indicates that the segregation between A and B compartments is less strict in *ΔWAPL* cells (Figures 6C and S4B).

To further investigate nuclear compartmentalization, we determined the association of chromatin with the nuclear lamina by performing DamID of Lamin B1 (Guelen et al., 2008). Lamina association correlates strongly with the B compartment, and similar to the A/B compartmentalization, we observed a decrease in the strong separation of lamina-associated and non-lamina-associated regions in *ΔWAPL* cells (Figures S4C and S4D). As a result, large lamina-associated domains (LADs) are fragmented into smaller LADs (Figures 6D and S4H).

When we quantified the regions that switched from A to B or vice versa, we found that more regions switched from B to A (46.0 Mb B to A versus 22.8 Mb A to B), and the compartment switches were strongly linked to a concomitant change in the association with the nuclear periphery (Figure 6E). Notably, the preferential B to A switch suggests that the active compartment is dominant over the inactive compartment. Interestingly, we found that a B to A switch often coincided with an increase in gene expression, whereas gene expression was not evidently affected for regions switching from A to B (Figure S4E). We propose that nuclear compartmentalization requires a certain degree of flexibility of chromosomes, and WAPL is required to limit the amount of cohesin on chromatin to provide this flexibility.

## Discussion

### Processive Loop Enlargement

Here, we provide key insight into the mechanism by which cohesin structures chromosomes. We find that the size of chromatin loops is under control of cohesin, and the dynamics of cohesin’s DNA entrapment state determines the length of these loops. Our findings indicate that the formation of chromatin loops involves a processive mechanism, and the duration with which cohesin embraces DNA determines the degree to which loops are enlarged. How then exactly does stable DNA binding by cohesin allow processive loop enlargement? How distal CTCF sites find each other in the nucleus is a major question. Diffusion is an unlikely scenario considering that CTCF sites almost exclusively loop together in a convergent orientation. If CTCF sites were to find each other by random diffusion, there would be no way to tell the orientation of the site, as the sites are often hundreds of kilobases apart and the chromatin fiber is very flexible. One would therefore assume that there must be a linear scanning mechanism that, by tracing the DNA, can communicate the orientation of the contacting CTCF sites.

One linear scanning model is the so-called “facilitated tracking model” in which RNA polymerase brings enhancers to promoters by transcribing all the way from enhancers to promoters (Blackwood and Kadonaga, 1998). As RNA polymerase would then maintain contact with the enhancer, this would result in the formation of a loop. In this model, cohesin could, for example, act as a clamp that holds together the loop after it has been formed. According to this scenario, stabilizing cohesin on chromatin should result in a relative increase of all loops that are also found in wild-type cells. However, in our *ΔWAPL* cells, we do not observe an increase in these “primary” loops, but specifically in “extended” loops.

**Figure 7:**
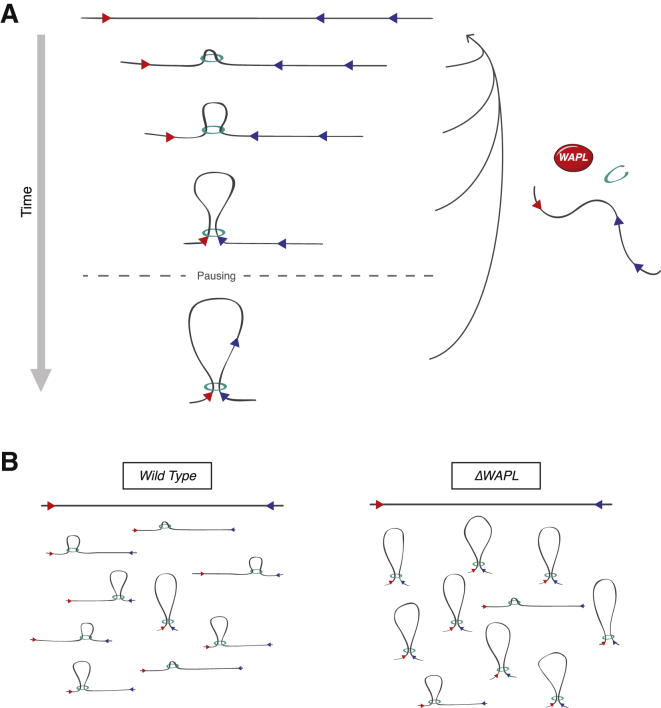
A Model Depicting the Role of WAPL in Chromosome Organization (A) WAPL restricts the extension of chromatin loops. We propose that CTCF sites are pausing sites for cohesin during the loop enlargement process. Cohesin complexes are depicted as green rings. Red and blue triangles denote forward and reverse CTCF sites, respectively. (B) Our data support the model that TADs in essence reflect populations of loops in the making between two given CTCF sites. WAPL through the constant disassembly of loops then allows TADs to be dynamic.

An alternative model would be that cohesin entraps a small loop in its lumen, and cohesin’s sliding down the base of this loop leads to loop enlargement. An important prediction of this “loop extrusion model” is that the stabilization of cohesin on DNA leads to longer loops. In this model, the looped DNA does, in fact, pass through cohesin during loop formation. Indeed, cohesin can slide along chromatin in vitro, and this requires the topological entrapment of DNA inside cohesin’s lumen (Davidson et al., 2016, Kanke et al., 2016, Stigler et al., 2016). Recent work in *B. subtilis* indicates that the SMC complex found in this species slides along DNA, and this movement coincides with the tethering together of DNA elements at the sites that it passes (Wang et al., 2017). Based on these different aspects, we favor the model that cohesin entraps a small loop in its lumen that is processively enlarged by cohesin’s sliding down the base of the loop (Figure 7A).

### To the Boundary and Beyond

The fact that in *ΔWAPL* cells loops can extend beyond CTCF sites indicates that CTCF boundaries are not absolute. In essence, there are two possible explanations for this phenotype. First, WAPL may affect the inherent boundary activity of CTCF sites. Earlier work has shown that the mutation or methylation of CTCF motifs can prevent CTCF binding, and this concomitantly abrogates the insulator function of these sites (Flavahan et al., 2016, Narendra et al., 2015). In *ΔWAPL* cells, however, CTCF’s binding to DNA is not evidently affected. This makes it unlikely that WAPL deficiency affects the boundaries themselves. Therefore, we favor the model that the boundary is not affected by WAPL deficiency, but rather that the number of attempts at passage by cohesin is larger in the absence of WAPL.

How CTCF acts as a boundary is unknown. It could, for example, be through physical obstruction of cohesin but also by creating a high-affinity site for cohesin. In both of these cases, an increase in the likelihood of passage could be explained by a longer residence time of cohesin on DNA in WAPL-deficient cells. If cohesin’s residence time on DNA then becomes considerably longer than that of CTCF, one would expect that cohesin pauses at CTCF sites until CTCF dissociates from DNA, after which cohesin can resume the loop enlargement process. If cohesin binds directly to CTCF, but the off-rate of this interaction is higher than that of cohesin’s DNA entrapment state, then cohesin can again proceed once it dissociates from CTCF. In both cases, CTCF sites merely are pausing sites for cohesin during the loop enlargement process (Figure 7A).

As the length of chromatin loops turns out to be under control of WAPL, this allows for the scenario that cohesin complexes in some instances are protected against WAPL to allow either the stabilization of loops or the further enlargement of loops. An important precedent for such protection is provided by the cohesin complexes that hold together the sister chromatids. Through the acetylation of cohesin’s SMC3 subunit, these complexes are rendered resistant to WAPL and thereby locked on the DNA from DNA replication until mitosis. The local and temporal protection against WAPL also plays an important role during mitosis and allows the WAPL-dependent removal of most cohesin complexes from chromosome arms but not centromeres that leads to the classical X-shape of mitotic human chromosomes (Haarhuis et al., 2014). Therefore, it is conceivable that cohesin-mediated looping is likewise controlled through protection against WAPL.

### WAPL and Illegal Loops

Our finding that WAPL deficiency also leads to the formation or maintenance of loops between incorrectly oriented CTCF sites indicates that WAPL not only restricts chromatin loop extension, but also enforces the “CTCF directionality looping rule.” This raises the interesting possibility that both correct and incorrectly oriented loops are formed in wild-type cells, but WAPL normally removes these “illegal” loops. If so, there may be a yet to be identified signal that allows WAPL to specifically remove cohesin from non-convergently oriented sites. One explanation could indeed be that cohesin is protected against WAPL when it loops together convergent CTCF sites.

We should emphasize that illegally oriented loops remain relatively rare, even in the absence of WAPL. We do find extended loops genome-wide in *ΔWAPL* cells. The latter phenotype, therefore, is unlikely to be caused by the former. The extended loops could, however, lead to illegal loops, as cohesin complexes could, in principle, collide during the loop enlargement process. Cohesin could hereby end up pausing at an illegal CTCF site that is occupied by another cohesin complex at the base of an adjacent legal loop. It remains an important question for the future how WAPL-mediated cohesin release enforces the specificity of chromosomal loops.

### Cohesin and Nuclear Architecture

Earlier studies have found that chromosome topology can be remarkably refractory to changes (Lin et al., 2012). For example, cohesin depletion does not evidently disrupt nuclear compartmentalization (Seitan et al., 2013). Our data are in correspondence with this report, and we would suggest that high levels of cohesin stably associated with DNA actually counteract compartmentalization, and WAPL-mediated cohesin turnover provides the necessary chromosomal flexibility for nuclear compartmentalization.

Global effects on chromosomal loops have also been challenging to achieve. Stimulation with tumor necrosis factor alpha (TNF-α) does not drastically influence chromosome topology, despite substantial changes in gene expression (Jin et al., 2013), suggesting that many regulatory interactions are pre-formed (de Laat and Duboule, 2013). Fundamentally different chromosome topologies have so far primarily been observed when comparing different developmental lineages (Dixon et al., 2015, Krijger et al., 2016).

We now find that removing either WAPL or SCC4 is sufficient to affect chromosome topology on a global scale, be it through the formation of longer or shorter loops, respectively. It turns out that the SCC2/SCC4 complex and WAPL are involved in a balancing act that controls chromosome architecture at many levels, from the extension of loops to macro-scale interphase chromosome architecture.

We should note that even though WAPL deficiency affects loop length genome-wide, only ∼1,000 genes are differentially expressed. Chromatin loops, therefore, only affect the expression of certain genes. We anticipate that specifically these genes are dependent on distal promoter-enhancer interactions. It remains an important question how WAPL, SCC2/SCC4, and cohesin act collectively to modulate specific changes in gene expression.

### SMC Complexes and Loop Formation

The macro-scale architecture of WAPL-deficient vermicelli chromosomes has a striking similarity to that of mitotic chromosomes (Figure 6A and Naumova et al., 2013). Both types of chromosomes harbor virtually no far-*cis* interactions, which presumably in both cases reflects the rigidity of these chromosomes. A fundamental difference, however, is that condensed mitotic chromosome arms are almost completely devoid of cohesin (Haarhuis et al., 2014), and mitotic condensation is, to a large degree, dependent on the condensin complex (Hirano, 2016). Cohesin and condensin are highly related SMC complexes that can entrap DNA inside their lumens (Cuylen et al., 2011, Haering et al., 2008). Another crucial shared feature of vermicelli and mitotic chromosomes is that both display an axis of SMC complexes at the heart of each chromosome that spans its entire length. In vermicelli chromosomes, this axis consists of cohesin complexes, while in mitotic chromosomes condensin has this axial localization. The vermicelli axial cohesin patterns in *ΔWAPL* cells may well be the consequence of loop extension. The formation of many long loops could thus lead to an axis of cohesin complexes that de facto are at the base of the loops.

The processive loop enlargement model was originally proposed as an explanation for how condensin drives mitotic condensation, and at the time, it was suggested that cohesin may act in a similar manner (Nasmyth, 2001). We now provide experimental evidence that cohesin indeed acts through the processive enlargement of chromatin loops, which in turn raises important questions regarding the mode of action of the condensin complex. Considering the large degree of conservation between these complexes and their similar distribution at chromosome axes, it is a likely scenario that they act in fundamentally the same manner. Both complexes harbor conserved ABC-like ATPase machineries. Whether these complexes provide topological anchors to allow the extension of DNA loops by other factors, or if their own enzymatic activities rather harbor a DNA extrusion activity is an important question for the future.

## STAR★Methods

### Key Resources Table

**Table:** 

REAGENT or RESOURCE	SOURCE	IDENTIFIER
**Antibodies**		

WAPL	Santa Cruz	Cat# A-7
SCC4	Abcam	Cat# Ab46906
SCC2 N-terminal	Santa Cruz	Cat# C-9
SCC2 C-terminal	Absea	Cat# Serum of KT55
HSP90	Santa Cruz	Cat# H-114
Actin	Santa Cruz	Cat# I-19
CDK4	Santa Cruz	Cat# C-22
SCC1	Millipore	Cat# 05-908
CTCF	Millipore	Cat# 07-72
SMC1A	Bethyl	Cat# A300-055a
IgG Rabbit	Sigma	Cat# I5006
Goat anti-Rabbit-PO	DAKO	Cat# P0448
Goat anti-Mouse-PO	DAKO	Cat# P0447
Rabbit anti-Goat-PO	DAKO	Cat# P0449
Rabbit anti-Rat-PO	Santa Cruz	Cat# Sc2006
Goat anti-Mouse-488	Mol probes	Cat# A11029

**Chemicals, Peptides, and Recombinant Proteins**		

Blasticidin S	Invitrogen	Cat# R210-101
Puromycin	Sigma-Aldrich	Cat# P7255
TRIzol	Invitrogen	Cat# 15596-018
Poly-L-lysine	Sigma-Aldrich	Cat# P8920
DAPI	Sigma-Aldrich	Cat# D9542
Fix buffer I	BD biosciences	Cat# 557870
Propidium Iodide	Cayman Chemical Company	Cat# 10008351
RNase A	QIAGEN	Cat# 1032722
Dynabeads M270 streptavidin-coated beads	Life technologies	Cat# 65305
Circligase II	Epicenter	Cat# CL9025K
Accuprime Taq HiFi	Life technologies	Cat# 12346-086
MboI	New England Biolabs	Cat# R0147L
Biotin-14-dATP	Life Technologies	Cat# 19524-016
DpnII	New England Biolabs	Cat# R0543L
Csp6I	Thermo Scientific	Cat# ER0211

**Critical Commercial Assays**		

TruSeq PolyA Stranded mRNA sample preparation kit	Illumina	Cat# RS-122-2103
HiSeq SR Cluster Kit v4 cBot	Illumina	Cat# GD-401-4001
Cleanpcr beads	CleanNA	Cat# CPCR-0050
KAPA Library preparation kit	Illumina	Cat# KK8234

**Deposited Data**		

Hi-C, 4C, DamID,RNA-seq, ChIP-seq, haploid screen	This paper	GEO: GSE95015

**Experimental Models: Cell Lines**		

HAP1	Carette et al., 2011	N/A
HAP1 *ΔWAPL*	This paper	N/A
HAP1 *ΔSCC4*	This paper	N/A
HAP1 *ΔWAPL /ΔSCC4*	This paper	N/A
HAP1 *SCC2inA*	This paper	N/A
HAP1 *ΔWAPL / SCC2inA*	This paper	N/A
HAP1 *SCC2 Δ2-10*	This paper	N/A
HAP1 *ΔWAPL / SCC2 Δ2-10*	This paper	N/A
Hek293T	ATCC	N/A

**Experimental Models: Organisms/Strains**		

**Recombinant DNA**		

pX330	Addgene	Cat# 42230
pDonorBlast	Blomen et al., 2015	N/A
pDonorPuro	Blomen et al., 2015	N/A
SCC1-GFP	Gerlich et al., 2006	N/A
Gene-trap plasmid	Jae et al., 2013	N/A

**Sequence-Based Reagents**		

Gene editing primers	Table S1	N/A
ChIP primers	Table S1	N/A
4C primers	Table S1	N/A
Hi-C primers	Table S1	N/A
Primers for LAM-PCR procedure	Blomen et al., 2015	N/A
Linker for ssDNA ligation	Blomen et al., 2015	N/A
SCC4 TARGETplus Smartpool	Dharmacon	Cat# L-031981-01-0005

**Software and Algorithms**		

LAS-AF FRAP-Wizard	Leica	N/A
Fiji	ImageJ	http://.imagej.net/welcome
Prism	GraphPad	www.graphpad.com
SoftWorx	Applied Precision	N/A
Crispr design	Crispr.mit.edu	N/A
Bedtools	42	N/A
Bowtie	Langmead et al., 2009	N/A
Bowtie2 v2.2.6	Langmead et al., 2009	N/A
Cutadapt	Martin, 2011	N/A
HiC-Pro v2.7.7	Servant et al., 2015	N/A
Juicer	Durand et al., 2016	N/A
HiCseg	Lévy-Leduc et al., 2014	N/A
peakC	This paper	https://github.com/deWitLab/peakC
Rank-product	Breitling et al., 2004	N/A
DESeq2	Love et al., 2014	N/A
HTSeq	Anders et al., 2015	N/A
Macs2 v2.1.1	Feng et al., 2012	N/A
R v3.1.X	https://www.r-project.org/	N/A
MEME suite v4.11.1	Grant et al., 2011	N/A

### Contact for Reagent and Resource Sharing

Further information and requests for resources and reagents should be directed to and will be fulfilled by the Lead Contact, Benjamin D. Rowland (b.rowland@nki.nl).

### Experimental Model and Subject Details

HAP1 cells (Carette et al., 2011) were cultured in IMDM (Invitrogen) supplemented with 10% FCS (Clontech), 1% Penicillin-Streptomycin (Invitrogen) and 1% Ultraglutamin (Lonza).

### Method Details

#### Gene Editing

CRISPRs targeting *WAPL* (5′-CACCGCGTTCCATAGTATCCTGTA-3′), *SCC4* (5′-CACCGTACGGGCCTCGATGCGCTG-3′), *SCC2inA* (5′-ATCCCCGCAAGAGTAGTAAT-3′), *SCC2 Δ2-10* CRISPR 1(5′-CCTGAACTAAGTACTTTTAT-3′) *SCC2 Δ2-10* CRISPR 2 (5′-GTTTATTCTTGATAGGTTTA-3′) were cloned into px330 (Addgene plasmid #42230). *ΔWAPL* and *ΔSCC4* HAP1 clones were generated by insertion of a Blasticidine or Puromycin cassette respectively, as previously described (Blomen et al., 2015). *ΔWAPL*/*ΔSCC4* cells were generated by knocking out *SCC4* in *ΔWAPL* cells. The parental HAP1 cells harbor mutant p53 (Ser214Gly). As we previously showed that p53 depletion allows proliferation of WAPL deficient cells (Haarhuis et al., 2013), we could efficiently generate *ΔWAPL* HAP1 clones. The *SCC2 Δ2-10* truncation was generated using a repair oligo (5′-AGCTGGCACCTGAACTAAGTACTTTACCTATCAAGAATAAACCATCAAAG-3′). pBabe was co-transfected (1:10 ratio to CRISPRs) to select for transfected cells by puromycin (2 ug/ml, Sigma-Aldrich) addition for two days.

#### Hi-C

Hi-C was performed essentially as previously described (Rao et al., 2014). Briefly, for each template roughly 10 million cells were crosslinked using 2% formaldehyde. In nucleus restriction was performed using MboI. Restriction overhangs were filled in with biotinylated nucleotides followed by blunt-end ligation. A streptavidin pull-down was performed to enrich for ligated sequences. Pulled-down DNA was end-repaired and an A-overhang was added. Sequencing adapters were ligated to the DNA samples to create sequencing libraries. Libraries were sequenced on a HiSeq 2500 (paired-end 100bp) and on a HiSeq X (paired-end 150bp). We created two templates for wild-type and the three mutant cell lines to control for differences in template generation.

**Figure S5:**
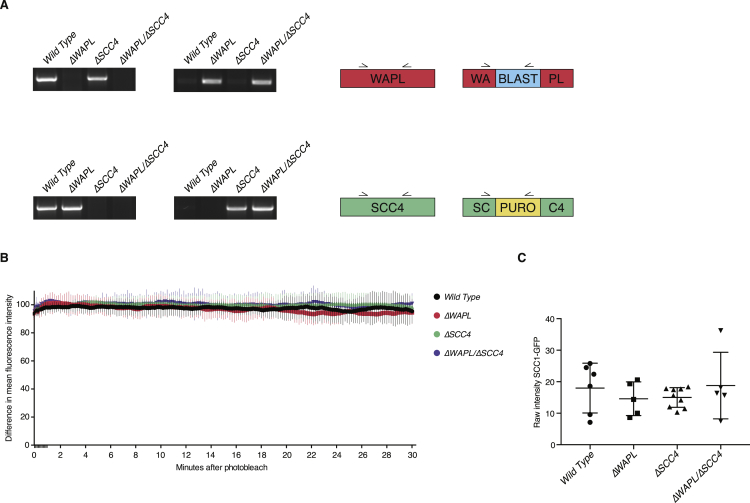
Genotype Analyses, Related to Figure 3 (A) Genotype analysis of the indicated cell lines. (B) Bleaching control for FRAP analyses. To control for bleaching during acquisition, a neighboring cell with similar SCC1-GFP expression was monitored (wild-type n = 7, *ΔWAPL* n = 6, *ΔSCC4* n = 10, *ΔWAPL/ΔSCC4* n = 6). (C) Raw intensity measurements of cells used for FRAP in Figure 3I. Each dot depicts the average intensity of 5 measurements before bleaching and corrected for the background signal. Line indicates the mean and the error bars indicate standard deviations.

**Figure S6:**
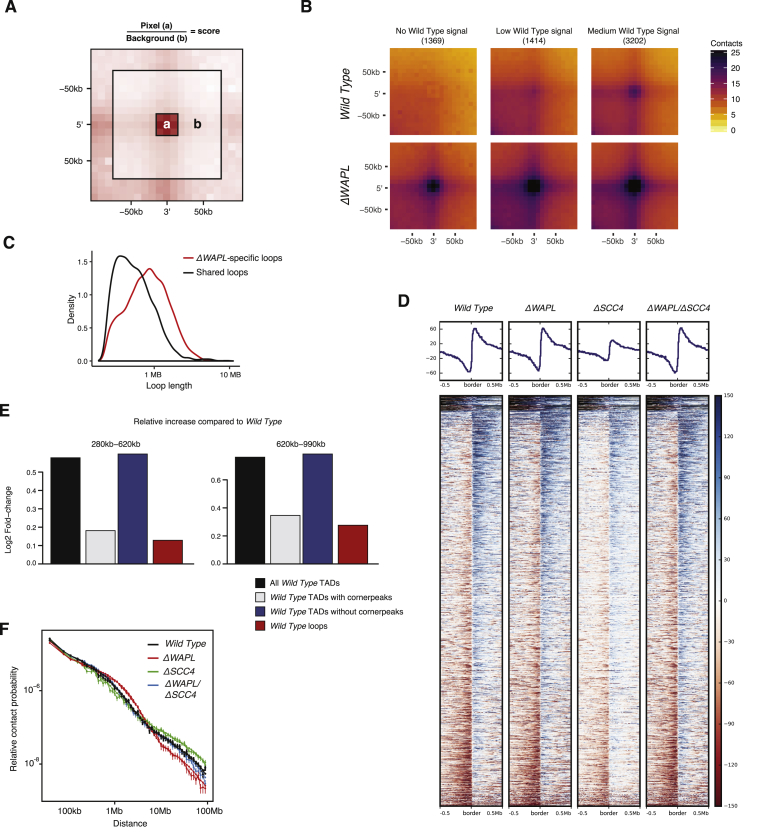
Extended Analysis of Hi-C Data, Related to Figures 5 and 6 (A) Loop-scores are computed by dividing the mean interaction score of the site (i.e., pixel) by the mean interaction score of the surrounding region. (B) *ΔWAPL*-specific loops are found by intersecting the *ΔWAPL* and wild-type loops. We determined the loop-scores of these sites in the wild-type data and stratified on relative enrichment of the pixel into three groups: no, weak and medium signal. (C) Length distribution of loops. Aspecific loops have a shorter mean length than *ΔWAPL*-specific loops. (D) Profile-plots and heatmaps of the DI-score alignment on wild-type TAD-borders, as depicted in Figure 5E. (E) TADs with cornerpeaks have a similar increase in signal to primary loops between wild-type and *ΔWAPL*. TADs without these cornerpeaks show a significant increase in signal at their peaks compared to primary loops of similar length (p = 5.5e-07). (F) Relative contact probability (RCP) plot of template replicates. Error bars depict the standard error of the mean.

**Figure S7:**
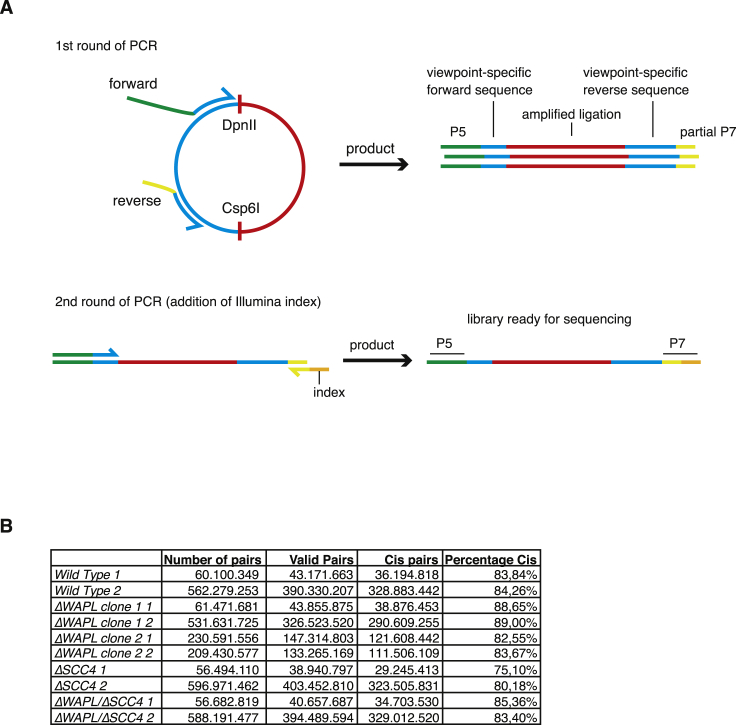
Nested 4C PCR Strategy for the Addition of Illumina Indexes and Hi-C Statistics, Related to Figure 2 (A) We have developed a nested PCR strategy for the generation of 4C libraries. The first round of PCR is an inverse PCR to amplify the fragments (red) ligated to the viewpoint (blue). The forward primer contains a viewpoint-specific sequence (which is used after sequencing to identify the viewpoint) and the Illumina P5 adaptor sequence. The reverse primer contains a viewpoint-specific sequence and a partial Illumina P7 adaptor. In the second round of PCR, the forward primer from the first round is reused and a universal reverse primer is used containing an Illumina index sequence. After amplification and clean-up this library is ready for sequencing. (B) Statistics on the number of read pairs, valid read pairs and percentage *cis* pairs. See also Table S1.

Raw sequence data was mapped and processed using HiC-Pro v2.7.7 (Servant et al., 2015). Data was mapped to hg19. Statistics on the number of read pairs, valid read pairs and percentage *cis* are summarized in Figure S5. Valid pairs files from HiC-Pro were transformed to juicebox-ready files using juicer-pre (Durand et al., 2016). We used HICCUPS v0.9 (Rao et al., 2014) to call loops at the following resolutions: 5kb, 10kb and 25kb. Raw loop calls were processed such that we merge flanking loops into a single loop.

#### TAD analysis

TADs were called using HiCseg (Lévy-Leduc et al., 2014). We used 10kb matrices from HiC-Pro as input, giving us a 10kb resolution for the TADs. At this resolution HiCseg becomes computationally intensive for a full chromosome. We therefore selected 15Mb submatrices from the chromosome, which were used as input for HiCseg. We chose the Poisson distribution (“P”) and the extended block diagonal model as the settings for the TAD calling and a maximum number of change-points of 50. To be able to also call TADs overlapping the border of two submatrices, we used 5Mb overlaps for the submatrices. For all subsequent TAD analyses we used the TADs that were identified in wild-type cells.

The directionality index (DI) was calculated as described (Dixon et al., 2012) on 10kb matrices using 2Mb windows up and downstream to calculate the directionality. To measure TAD integrity, we aligned the DI for all four cell lines on the 5′ TAD border. We determined the intra and interTAD contact frequency as follows: given two TADs *A* and *B* that span windows *a’* to *a”* and *b’* to *b”*, respectively and an nxn contact matrix *C*, where n is the number of windows in the genome. The intraTAD scores for A are calculated as follows:

$$\sum_{i=a^{'}}^{a^{''}}\sum_{j=i}^{a^{''}}C_{i,j}\;$$

The interTAD scores between A and B are calculated as follows:

$$\sum_{i=a^{'}}^{a^{''}}\sum_{j=b^{'}}^{b^{''}}C_{i,j}\;$$

We calculate interTAD scores for all TADs with the 10 flanking TADs. Note that in this analysis TADs smaller than 200kb are left out. The interTAD scores of neighboring TADs are used to determine the link between differential expression and chromosome organization.

#### 4C

4C was performed essentially as previously described (van de Werken et al., 2012). Similar to the Hi-C we created two templates for each cell line and used roughly 10 million cells to create a 4C template. We used DpnII as the first restriction enzyme and Csp6I as the second restriction enzyme. For every viewpoint we performed a two-step PCR reaction using viewpoint specific primers in the first round and universal index primers in de second round (see Figure S4 for more details). The forward (‘reading’) primers contained the entire P5 sequencing primer. The reverse primer contained a viewpoint specific sequence and 30nt of the P7 sequencing primer in the initial PCR round. Per viewpoint 5 initial rounds of PCR of 20 cycles were performed on 100ng of 4C template. In a second round of PCR we combined 5 ul of the initial 5 PCR volumes and ran a PCR using the forward adapters from the initial round of PCR and barcoded universal adapters. 4C libraries were sequenced on a HiSeq 2500 (single end 65).

Reads for every sample were demultiplexed and trimmed based on the reading primer adding GATC to the start of the sequence. Trimmed reads were mapped to the genome using bwa bwasw (Li and Durbin, 2010). Reads that did not start at a DpnII restriction site were filtered out as they probably do not represent ligation events. Non-unique fragments and fragments created by the digestion of two DpnII sites (“blind” fragments) were also filtered out.

Peak calling was performed using peakC (https://github.com/deWitLab/peakC). The main assumption in our 4C analysis is that the contact frequency decreases monotonically with the distance. We use monotonic regression to model the background contact frequency. Because the background contact frequency can differ between the region upstream and downstream of the viewpoint we model these independently. In order to identify regions that are significantly contacted by the viewpoint we have developed a statistical framework that enables the integration of multiple 4C experiments. For every experiment the background model is calculated. Next we calculate both the ratio (*R*) and the difference (*Δ*) between the fragment end coverage and the expected background coverage for this fragment end. We use both the ratio and the difference between the actual value and the expected value to be able to include small values of *R* that yield large differences in Δ and vice versa to prevent small differences in Δ that can give rise to large differences in *R*.

For the combining of multiple experiments we make use of the rank-product (Breitling et al., 2004). Originally developed for the identification of differentially expressed genes, rank products are an intuitive, non-parametric way of combining independent experiments. A non-parametric analysis is appropriate since the distributions of R and Δ do not follow a normal distribution. We ranked both *R* and *Δ* (from high to low) and subsequently ranked the average of both ranks. This gives us a rank for every fragment in each experiment. To combine the experiments we calculate the product of the rank for every fragment. To approximate a p value we make use of the Gamma distribution (Koziol, 2010):

$$P=1-\text{Γ}-\text{ln}\frac{r}{{\left(n+1\right)}^{k}}$$

Although, an exact calculation of the p value has been proposed (Eisinga et al., 2013), for larger samples this is computationally prohibitive.

#### CTCF-associated loops

Based on the ChIPseq data of CTCF we can annotate the loops identified by HICCUPs. Loop anchors were intersected with a list CTCF peaks that were found in wild-type or *ΔWAPL* cells or both. CTCF peaks were intersected with CTCF motifs (MA0139.1 from JASPAR CORE 2014 (Mathelier et al., 2014) using FIMO (Grant et al., 2011) to annotate the orientation of the CTCF binding site. To quantify the CTCF directionality of chromatin loops, we only selected those loops that have a unique orientation.

#### Antibodies

Western Blots were performed using the following antibodies: WAPL (Santa Cruz, A-7), SCC4 (Abcam, ab46906), SCC2 N-terminal (Santa Cruz, C-9), SCC2 C-terminal (Absea, serum of KT55), HSP90 (Santa Cruz, H-114), CDK4 (Santa Cruz, C-22) and Actin (Santa Cruz, I-19). All antibodies were used at 1:1000 dilution. Secondary antibodies Goat anti-Rabbit-PO, Goat anti-Mouse-PO, Rabbit anti-Goat-PO (DAKO) and Rabbit anti-Rat-PO (Santa Cruz, sc2006) were used at 1:2000 dilution. For immunofluorescence we used the SCC1 (Millipore, 05-908) antibody in a 1:100 dilution. CTCF (Millipore, 07-72), SMC1A (Bethyl, A300-055a) and IgG Rabbit (Sigma, I5006) were used for chromatin immuno-precipitations.

#### Microscopy

For FRAP assays, cells were grown on a LabTekII-chambered cover glass (Thermo Scientific Nunc) coated with poly-L-Lysine (Sigma). Two days before imaging cells were transfected with SCC1-GFP (Gerlich et al., 2006). Prior to imaging the medium was changed to pre-warmed Leibovitz L-15 medium (Invitrogen). Images of G1 cells were captured on a Leica SP5 confocal microscope with a 63x/1.4 oil immersion objective using the LAS-AF FRAP-Wizard. Half of the nucleus was photobleached 5 times with 100% transmission of a 488 nm laser, followed by time-lapse imaging (10 times every 1,2 s, followed by 180 times every 10 s). Measurements were taken in user-defined regions and adjusted by hand for nucleus movements. Bleaching by imaging was monitored by quantifying the mean GFP intensity of a non-bleached cell. Noise was reduced by performing a rolling average (averaging five frames at a time). Recovery was quantified by the difference in mean intensity in bleached and unbleached regions after background correction. Stills were taken of a merge of five frames. Brightness and contrast between movies was adjusted for visualization purposes.

For immune-fluorescence, cells were grown on 9 mm coverslips coated with poly-L-Lysine (Sigma) and pre-extracted using 0.1% Triton/PBS for exactly 1 min and subsequently fixed with 3.7% PFA/PBS for 7 min. Samples were blocked with 4% BSA/PBS for 1 hr at room temperature and incubated with the SCC1 antibody in 2% BSA/PBS overnight at 4°C. After three PBS washes, samples were incubated for 1 hr with secondary antibody and 1 μg/ml 4’,6-diamidino-2-phenylindole (DAPI). Cells were mounted onto glass slides using Prolong antifade gold (Invitrogen). Imaging for chromatin-bound cohesin quantifications was performed using a DeltaVision Elite system (Applied Precision) equipped with 63x/1.42 oil lens. Z stacks of the whole cell were deconvoluted using SoftWorx (Applied Precision) and quantification of the mean intensity of SCC1 in the DAPI region was performed using an in house written ImageJ macro. Immunofluorescence images were taken on a confocal laser-scanning microscope (Leica), equipped with HCX Plan-Apochromat 63x/1.32 oil lens using LAS-AF Software. Brightness levels of SCC1 images were equally adjusted.

Chromosome spreads were performed as described (Elbatsh et al., 2016). Spreads with 3 or more chromosomes that lost their centromeric constriction were quantified as harboring a railroad phenotype.

#### RNA-seq

Total RNA from cultured cells was extracted using TRIzol reagent (Invitrogen). Strand-specific libraries were generated using the TruSeq PolyA Stranded mRNA sample preparation kit (iIlumina). In brief, polyadenylated RNA was purified using oligo-dT beads. Following purification, the RNA was fragmented, random-primed and reserve transcribed using SuperScript II Reverse Transcriptase (Invitrogen). The generated cDNA was 3′ end-adenylated and ligated to Illumina Paired-end sequencing adapters and amplified by PCR using HiSeq SR Cluster Kit v4 cBot (Illumina). Libraries were analyzed on a 2100 Bioanalyzer (Agilent) and subsequently sequenced on a HiSeq2000 (Illumina). We performed RNaseq alignment using TopHat 2.1.1 (Kim et al., 2013). Differentially expressed genes were called with DEseq2 (Love et al., 2014), with an adjusted p value threshold of 0.05.

#### Variant calling

SomaticSniper v1.0.5 was used to call *ΔWAPL*-specific mutations using the raw Hi-C reads of wild-type and *ΔWAPL* (Larson et al., 2012). To validate these variant calls, we used GATK Mutect2 (McKenna et al., 2010) and variants were annotated with the Ensembl Variant Effect Predictor. Using these annotations, we found no exonic moderate- or high-impact mutations. To further validate these findings, we used the RNA-seq data for finding *ΔWAPL*-specific mutations. Variants of wild-type, *ΔWAPL*, *ΔSCC4* and *ΔWAPL/ΔSCC4* were called using the best practices of GATK (DePristo et al., 2011). In brief, mapping was performed with STAR (Dobin et al., 2013) and variant calling was performed with HaplotypeCaller (McKenna et al., 2010). We found no *ΔWAPL*-specific mutations.

#### ChIP-seq

Chromatin immune precipitations were performed as described (Schmidt et al., 2009) with slight modifications. Cell lines were grown until 70% confluency and 5 × 10^∧^7 cells were used per ChIP. Cells were fixed in 1% formaldehyde; 50mM HEPES-KOH; 100mM NaCl; 1 mM EDTA; 0,5 mM EGTA. Cell lysis was performed in LB1 buffer with final pH 7,5 (50 mM HEPES-KOH; 140mM NaCl; 1mM EDTA, 10% Glycerol, 0.5% NP-40; 0,25% Triton X-100; Proteinase inhibitor) for 20 min at 4°C. Subsequently, nuclei were lysed using LB2, pH 8,0 (10mM Tris-HCl; 200 mM NaCl; 1mM EDTA; 0,5mM EGTA; Proteinase inhibitor) for 10 min at 4°C. Pellets were resuspended in LB3 pH8,0 (10mM Tris-HCl; 100mM NaCl; 1 mM EDTA; 0,5 mM EGTA; 0.1% Na-Deoxycholate; 0.5% N-lauroylsarcosine; Proteinase inhibitor). Cross-linked chromatin was sheared (400-800 bp) using a Covaris S2 with Tube and Caps (Covaris, 520048), using the following settings: duty cycle: 10%, intensity: 4, cycles per burst: 200 time 40 s with 20 cycles. Chromatin precipitation was performed overnight using antibody-bound (CTCF 5 μl, SMC1 10 μl, Igg 10 μL per IP) proteinA coupled DynaBeads (Invitrogen). Elution and decrosslinking was performed overnight at 65°C in EB pH 8,0 (50mM Tris-HCl; 10mM EDTA; 1% SDS). Samples were treated with Proteinase K and RNaseA for 2 hr. DNA was isolated using phenol extraction and ethanol precipitation. Library preparation was done using a KAPA Library preparation kit using the manufacturer’s protocol. Products were sequenced using an Illumina HiSeq 2500 (single end 65bp).

Reads were mapped to hg19 using Bowtie2.1.0 (Langmead and Salzberg, 2012) with default settings. Regular peak calling was performed using MACS2 (v2.1.1) (Feng et al., 2012) using IgG as control. Venn diagrams were generated using the R package vennEuler. Heatmaps were generated for the union of SMC1 peaks in the wild-type and ΔWAPL cell line using seqMiner (Ye et al., 2011). Heatmaps were clustered with k-means clustering.

#### Synthetic Viability Screens

Genes required for viability of three independent *ΔWAPL* cell lines were profiled as previously described in detail (Blomen et al., 2015). In short, gene trap retrovirus was produced in HEK293T cells by transfection of the packaging plasmids Gag-pol, VSVg, and pAdvantage in addition to the previously described gene trap plasmid (Jae et al., 2013). Retrovirus was harvested twice a day for a period of at least three days and pelleted by centrifugation at 22.000 rpm for 2 hr at 4C in a SW28 rotor. One day prior to infection, *ΔWAPL* cells were seeded with 40 million cells in a single T175 flask. Infection occurred over the consecutive days using the pelleted gene trap retrovirus in the presence of 8 ug/ml protamine sulfate. The mutagenized *ΔWAPL* cells were passaged for 10-12 days following the last infection with the mutagen, collected following dissociation using trypsin-EDTA by pelleting, and fixed using fix buffer I (BD biosciences). In order to minimize confounding from diploid cells carrying heterozygous mutations, the fixed cells were stained using DAPI for DNA content and sorted for haploid cells in the G1 phase of the cell cycle on an Astrios Moflo. Genomic DNA was isolated from 30 million sorted cells using a DNA mini kit (QIAGEN) with the lysis step occurring overnight at 56°C for de-crosslinking.

The gene trap insertion sites were amplified using a LAM-PCR procedure as described (Blomen et al., 2015). Using a biotinylated primer in the gene trap cassette, single-stranded DNA (ssDNA) products were generated for 120 cycles, captured on magnetic beads, a pre-adenylated ssDNA linker ligated to the 3′ end, and a final round of exponential amplification using a primer containing Illumina sequencing compatible overhangs at the end of the LTR and in the ssDNA linker. Following PCR purification (QIAGEN) samples were sequenced on a HiSeq 2000 or HiSeq 2500 (Illumina). Insertion sites were mapped by aligning the deep sequencing reads to the human genome (hg19) using bowtie (Langmead et al., 2009) allowing for a single mismatch. Reads from the HiSeq 2500 (65bp) were cropped to 50 bp similar to the output for the HiSeq 2000 runs. Unique aligned reads were subsequently assigned to Refseq gene coordinates using Bedtools (Quinlan and Hall, 2010). Overlapping gene regions on opposite strands were disregarded (since orientation bias in that region is not readily interpretable), while of genes with overlapping regions on the same strand the names were concatenated. For each replicate a binomial test for the distribution of sense and antisense orientation insertions was performed. Genes of interest reported here were examined for the percentage of sense orientation insertions in both the three *ΔWAPL* cell lines generated for this manuscript and four previously reported wild-type HAP1 datasets (Blomen et al., 2015) (NCBI SRA accession number SRP058962).

#### Proliferation assays

For 3T3-like proliferation curves, 150.000 cells were seeded in 6 well plates and counted and reseeded at the same density every 3 days. For Colony Formation assays cells were seeded at 30,000 cells per well on a 48- well plate, transfected with siRNAs targeting SCC4 (10 μM ON-TARGETplus Smartpool) or mock control, and grown for 5 days. Plates were washed with PBS, fixed for 10 min using 96% methanol and stained with 0,25% crystal violet.

#### Compartment scoring

To segregate A and B compartments we performed eigenvalue decomposition of the Hi-C correlation matrix (Lieberman-Aiden et al., 2009). We used the Homer package (v4.8) to perform PCA analysis. The resolution was set to 100kb and the window size to 200kb. In order to identify genomic regions that robustly switch from A to B or vice versa we calculated the following threshold: we determined the range of the 15-85 percentile in the wild-type dataset and divided this by 2. We selected those regions as switchers where the absolute difference between the eigenvectors exceded this threshold and where the eigenvector switched from positive to negative or from negative to positive between the two conditions.

#### DamID

DamID was performed as described (Guelen et al., 2008), with the following modifications. In brief, 300.000 cells were plated per 6 well and transduced with virus encoding either DAM only or Lamin-DAM. Before harvesting cells were washed 4x with PBS. Genomic DNA isolation was performed using the Isolate II Genomic DNA isolation kit using the manufacturer’s protocol. Subsequently DNA was digested for 4 hr at 37°C by DpnI. Adapters were ligated overnight at 16°C and products were amplified using by PCR. DNA was isolated using a QIAquick PCR Purification kit and sheared using the Covaris S2 instrument using the following settings: duty cycle: 10%, intensity: 5, cycles per burst: 200, time: 45 s to create pieces between 300-800 bp. Library preparation was done using a KAPA HTP Library preparation kit according to the manufacturer’s instructions. Libraries were quantified on an Agilent Bioanalyzer and sequenced using an Illumina HiSeq2000 machine.

DamID profile analyses were performed as described previously (Pindyurin et al., 2016). Briefly, reads from Lamin-Dam and Dam-only were processed by removing the adaptor sequences: GGTCGCGGCCGAG or CTAATACGACTCACTATAGGGCAGCGTGGTCGCGGCCGAG using cutadapt (Martin, 2011). Then, the reads were mapped against the human reference genome hg19 using bowtie2 (Langmead and Salzberg, 2012) with default parameters (except for–local and –k 3). We discarded reads that mapped to multiple locations. Then, we calculated the coverage of reads per GATC fragment using the program htseq-count (Anders et al., 2015). GATC fragments were obtained by digesting in-silico the reference genome at every GATC motif with in-house scripts. We excluded un-mappable regions (defined by wgEncodeCrgMapabilityAlign36-mer) bigger than 5Kb from the GATC fragments. To reduce noise, we concatenated GATC fragments in 50Kb windows, with each 50Kb-extended GATC fragment starting and finishing in a GATC motif. The DamID profiles were calculated as the log2 ratio of the coverage of reads in the 50Kb-extended GATC fragments between Lamin-Dam and Dam-only. To define LADs, we concatenated adjacent 50Kb-extended GATC fragments enriched in reads in the Lamin-Dam over the Dam-only. We allowed the extension of LADs over GATC fragments not enriched in Lamin-Dam reads if their total amount of base pairs was smaller than the 20% of the length of the LAD.

#### Aggregate Peak Analysis

To appreciate the genome-wide effects of deleting one or more factors on the 3D genome confirmation we performed aggregate peak analysis (Krijger et al., 2016, Rao et al., 2014). In this analysis we calculate the average contact frequency over a given set of 2D genomic coordinates, such as loops or TAD corners. For a given 2D genomic coordinate we select a square region flanking the coordinate 100kb upstream and 100kb downstream. As a result we get a 210kb by 210kb, or 21x21 submatrix considering we use a 10kb resolution. We average all the submatrices, which results in an average contact profile over a given set of 2D coordinates (e.g., loops).

In our analyses we make a distinction between primary loops and extended loops. Primary loops are the loops that are called in the wild-type dataset (see above) and are formed between a 5′ loop anchor and a 3′ loop anchor. Extended loops were generated by combining a 5′ anchor of loop A with a 3′ anchor of loop B, when the 3′ anchor of loop B lies beyond the 3′ anchor of loop A and only when the 5′ anchor of loop B is not within 30kb of the 5′ anchor of loop A (i.e., they do not have the same 5′ anchor).

For TADs we do an aggregate TAD analysis. Which is similar to APA, but rather than selecting a 2D coordinate, we select entire TADs. In addition to the TAD we extend the position of the 5′ and 3′ TAD borders by 50% of the size of the TAD. Because TADs vary in size we resize the submatrices to 100x100. We subsequently average over all the submatrices.

### Quantification and Statistical Analysis

Statistical details can be found in the figure legends, or in the Method Details section.

### Data and Software Availability

Software used for Peak Calling in 4C can be found at: https://github.com/deWitLab/peakC

The accession number for the data for Hi-C, 4C, DamID, RNA sequencing, ChIP sequencing and Haploid Genetic Screens reported in this paper is NCBI GEO: GSE95015.

## Author Contributions

J.H.I.H., V.A.B., M.A., M.S.v.R., P.H.L.K., and H.T. performed experiments. R.H.v.d.W., E.d.W., V.A.B., and J.O.Y.-C. did computational analyses. R.H.M. provided laboratory infrastructure. B.D.R., E.d.W., T.R.B., and B.v.S. supervised the project. B.D.R., E.d.W., J.H.I.H., and R.H.v.d.W. wrote the manuscript with input from all other authors.

## Supplemental Information

Table S1. Oligonucleotides Used in this Study, Related to the STAR Methods
